# Relationship between social support and schizophrenia relapse among patients with schizophrenia on follow-up at Amanuel Mental Specialized Hospital, Addis Ababa, Ethiopia: A case-control study

**DOI:** 10.3389/fpsyt.2022.980614

**Published:** 2022-11-24

**Authors:** Tinbete Samuel, Kabtamu Nigussie, Yohannes Mirkena, Telake Azale

**Affiliations:** ^1^Department of Psychiatry, School of Nursing, College of Medical and Health Sciences, Hawasa University, Hawasa, Ethiopia; ^2^Department of Psychiatry, School of Nursing and Midwifery, College of Health and Medical Sciences, Haramaya University, Harar, Ethiopia; ^3^Department of Psychiatry, School of Medicine, College of Medical and Health Sciences, University of Gondar, Gondar, Ethiopia

**Keywords:** schizophrenia, relapse, social support, association, follow up, Ethiopia

## Abstract

**Background:**

Severe mental illnesses affect about 4% of the adult population worldwide. The illness is highly related to the relapse rate and can cause cognitive, social, and functional deterioration in patients. While there are some factors that are known to be protective of the occurrence of relapse in schizophrenia, having good social support is found to be one of the strongest factors. Therefore, the aim of this study was to identify the association between relapse and poor social support in patients with schizophrenia.

**Methods:**

With unmatched case-control (case—patients with schizophrenia with poor social support and controls—patients with schizophrenia with good social support), the study included 408 patients with schizophrenia on a follow-up at Amanuel Mental Specialized Hospital from March to May; 2020. The convenience sampling technique was used to draw the participants. Social support was assessed by the Oslo social support scale (Oslo-3), whereas Family Emotional Involvement and Criticism Scale and Medication Adherence Rating Scale-10 (MARS-10) were used to assess the emotion expressed and adherence to medication, respectively. Epidemiological Information (Epi Info) version 7 was used for data coding and entry, which were then exported to the Statistical Package for Social Sciences version 22 for analysis. Unconditioned binary logistic regression analysis and bivariate correlation analysis were carried out.

**Results:**

Out of 408 samples, 396 participants, of which 194 were cases and 202 were controls, were included in the study with a response rate of 97.06%. The mean and standard deviation (SD) age of the participants was 36.06 ± 9.842 years. From 194 cases, 61.1% of them were reported to have poor social support. In multiple logistic regression, only social support was found to be statistically significant [*p* < 0.001, adjusted odds ratio (AOR) = 3.102, confidence interval (CI) (95%) = (1.985–4.848)]. Hence, the odds of having relapse were 3.102 higher in those who have poor social support when compared to those with good social support. Emotional involvement, financial problems, and stressful life events were found to indirectly affect relapse by having a negative correlation with social support.

**Conclusion:**

The present study showed that there was a relationship between relapse and social support, and this indicates that social support can be a good predictor of schizophrenia relapse. The caregivers and clinicians of the patients should increase their support to the patients, while hospitals with mental health services have to encourage formation of better social support for the patients, including psychosocial interventions which will be performed to enhance the social support system.

## Background

Severe mental illnesses are explained by the deterioration of emotional, social, and economic statuses of an individual and also have a high risk for the development of physical illness (1).

According to the World Health Organization (WHO), more than 450 million people suffer from mental disorders worldwide and were estimated to account for 15% of disabilities around the world in 2020 (2, 3). Severe mental illnesses affect more than 4% of the adult population and are the main causes of cognitive, emotional, and behavioral disturbances (3–5). These disorders include schizophrenia, bipolar disorder, and major depressive disorder, which account for three of the six leading causes of years with disability (4, 5).

Schizophrenia is one of the most disabling and chronic mental disorders that affect more than 20 million people worldwide. Patients commonly experience perception without stimuli and fixed false beliefs. It affects their thinking, perception, emotion, language, sense of self, and behavior and functioning in social, educational, and occupational aspects (6).

Relapse refers to the reemergence, worsening of symptoms, or hospital readmission in the past 6 months and receiving more intensive case management or medication change (7). Schizophrenia is highly related to the repeated reemergence of symptoms (more than 50% of the patients) after remission globally, and every time the symptoms reoccur, the harder it will be to treat them. Relapse of symptoms not only does mean that there will be a higher chance of having a lifelong illness but also indicates its association with a higher risk of hospitalization, cost, and deterioration of the individual (6, 8–10). A systematic review showed that loneliness and perceived poor social support were related to poor outcomes in mental health problems such as schizophrenia, bipolar I disorder, depression, and anxiety disorder (11).

Studies done in Ethiopia showed a range of patients with schizophrenia, 24.6–87.69%, who have had a history of relapse through the course of the disease (7, 9, 12).

Studies also showed that factors, like poor social support, medication non-adherence, substance use, and stressful life events, are commonly associated with symptom relapse in patients with schizophrenia (5, 12, 13).

Social support is the physical and emotional comfort given to the affected people by their family, friends, coworkers, or others. It is possible only when people are acknowledged as a part of the community that loves and cares for them and values and respects them. Social support comes in four main types: emotional support, practical help, sharing points of view, and sharing information. It plays an important role in maintaining mental health, but mental health problems can affect social support, hence aggravating the feeling of loneliness and more mental health problems (14).

Even though literature suggests that having good social support is a form of protection against mental illnesses (14), there is also evidence to suggest that there is poor social interaction and support, and high stigmatization by society, of patients with SMI (Severe Mental Illness), especially with that of patients with schizophrenia due to an unexpected fluctuation of symptoms and a higher incidence of relapse, which also contribute to the poor outcomes of the disease process (5, 15, 16).

Poor social support is also indirectly associated with a lack of supervision and difficulty in accessing the medication prescribed, which leads to medication non-adherence, which is considered to be the highest risk for relapse in individuals with schizophrenia (13). As stated ealier, good social support can decrease the risk of relapse, however, there is no evidence of the association between social support and schizophrenia relapse in Ethiopia. Therefore, this study attempted to fill this information gap by identifying the association between relapse and poor social support in patients with schizophrenia and to provide important recommendations to plan important intervention programs that improve the early detection and management of schizophrenia relapse and determines poor social support among patients with schizophrenia.

## Methods and materials

### Study design, setting, and period

An institutional-based unmatched case-control study was conducted at Amanuel Mental Specialized Hospital, which is located in Addis Ababa, Ethiopia from March to May 2020. Amanuel Mental Specialized Hospital (AMSH), according to the hospital's Communication Directorate's report; the hospital was established by the Italian invaders in 1930 to serve as a medical setup for the native population. It has been serving as the only public specialized psychiatric hospital since 1948 under the Ministry of Health. The service was given by foreign psychiatrists from Russia, Bulgaria, Cuba, and Yugoslavia until it was overtaken by Ethiopian psychiatrists and other psychiatry professionals. The hospital has 235 beds in 14 wards and 2 emergency rooms for inpatient services such as general adult psychiatry, addiction, forensic, and Clozapine treatment services. It has 24 outpatient clinics that also include medical, maternal and child, and Assisted Reproductive Technology (ART) clinics.

### Eligibility criteria

All adult (age≥18 years) patients with a Diagnostic and Statistical Manual of Mental Disorders Version 5 (DSM-5) diagnosis of schizophrenia, who had a follow-up at the Amanuel Mental Specialized Hospital from second visit and above during the study period, were included in the study. Patients who were newly diagnosed patients with a DSM-5 diagnosis of schizophrenia, those with a known comorbid psychiatric condition, and those who had a critical health condition and were unable to communicate properly were excluded from this study.

#### Sample size determination and sampling procedure

The sample size was calculated with the help of Epi Info version 7.0 software, based on Kelsey's formula


$$\begin{array}{l} n_{1}=\frac{{\left(Z_{\frac{\alpha }{2}}+Z_{1-\beta }\right)}^{2}\;\times p\;\times \left(1-p\right)\times \left(r+1\right)}{{r\times \left(p_{0}-p_{1}\right)}^{2}}{\;n}_{2}=rn_{1}, \end{array}$$

where z_**α/2**_ refers to the confidence interval (CI), which is assumed to be 95%, Z_1−__β_ refers to power, which is assumed to be 80%, the proportion of cases is assumed as P0 and the proportion of controls as P1 (12), OR refers to the odds ratio, which is assumed to be 2, and r refers to the ratio of control to cases, which is 1:1 for this study. Therefore, the total calculated sample size was 408 with 204 cases and 204 controls, where cases were those patients with schizophrenia who have poor social support and the control group includes patients with schizophrenia with good social support. Participants were selected for the study with the help of a consecutive sampling technique. Eligible and willing participants were taken based on their availability or visit to the outpatient department, and samples of both case and control were taken until the desired sample size was attained.

### Data collection procedure and instruments

Data were collected using an structured interview questionnaire and a review patient's chart. The questionnaire contains six parts, which are socioeconomic characteristics of the patients adapted and modified from reviewing similar literature, clinical, psychosocial, and behavioral factors.

The social support was assessed by using the Oslo social support scale (Oslo-3). It covers unique fields of social support by way of measuring the quantity of people the respondent feels close to, the interest and subject shown with the aid of others, and the ease of obtaining practical assistance from others. Although the internal consistency of items on Oslo-3 according to one study done in Nigeria was the Cronbach's alpha of 0.5, it is a widely used tool to measure social support and validated in Ethiopia. The scale has three items assessing social support and is rated out of 14. The Oslo-3 scores ranged from 3 to 14, with a score of 3–8 for poor support; 9–11 for medium support; and 12–14 for strong support. This study used a cut-off point of ≥ 9 out of 14 to be categorized as having strong social support (17–19). In this study, the internal consistency of Oslo-3 was a Cronbach's alpha of 0.72.

Medication adherence was assessed by using the Medication Adherence Rating Scale-10 (MARS-10), which is used to assess medication adherence in some studies in Ethiopia. It has 10 items under it, which are answered with a simple yes or no. Patients who responded “no” for questions 1–6, 9, and 10 and “yes” for questions 7 and 8 were considered compliant (12, 20, 21). In this study, the internal consistency of MARS-10 was a Cronbach's alpha of 0.75.

The Family Emotional Involvement and Criticism Scale (FEICS) having 14 questions with a score ranging from 0 to 4 in each question was adapted and used as a tool to assess the expressed emotion. The tool has two scales, one assessing perceived criticism and the other assessing emotional involvement. Each scale has a score ranging from 0 to 28 and has to be considered as having a high family emotional involvement, and perceived criticism of the participants must have a score of 28 for both scales. Stressful life event was assessed using a list of life-threatening experiences (LTE) questionnaire, which has 12 items listing experiences which are assessed for stressful experience in the past 12 months, and if a participant answered yes even for a single question, he/she is considered to having a stressful life experience (22–24). The internal consistency of FEICS in this study was a Cronbach's alpha of 0.71). At the end of the questionnaire, the participants were categorized as relapse or none relapse by a single question “have you ever had reemergence or worsening of symptom or been rehospitalized in the past 1 year,” which will be filled by the supervisor to assign the participants as case or control.

### Data quality control

Data collectors and supervisors were trained for 3 days on the data collection approach of the study. The questionnaire was translated into the local language Amharic by a language expert and backtranslated into English by another person to check for consistency. A pretest was conducted on 5% of the sample size at Yekatit 12 Hospital to see the applicability of the instruments, and the feedback was incorporated into the final tool to improve the quality. The result was not included in the results of this study's finding. Supervision was done by the supervisor and principal investigator throughout the data collection period and checked daily for completeness and consistency of filled questionnaires.

### Data processing and analysis

All returned questionnaires were checked for completeness and consistency of responses manually, were coded, entered into EPI Info version 3.1 for data cleaning, and then exported to (Statistical Package for Social Sciences) SPSS version 22.0 for analysis. Descriptive statistics was done to assess the social support in both cases and controls, and for assessing relationships, a chi-square test and binary logistic regression analysis were carried out. Confounders were controlled by using multiple logistic regression analysis. In addition, a bivariate correlation was done to show associations between social support and other relapse determinants.

### Ethical consideration

The studies involving human participants were reviewed and approved by the Institutional Health Research Ethics Review Committee of the College of Medicine and Health Science, University of Gondar. The formal letter of permission and support was submitted to the research ethical committee of the Amanuel Mental Specialized Hospital, and ethical approval was obtained to carry out the data collection. The procedure and purpose of the study were thoroughly explained to each participant, and oral and written informed consent with a signature from the participants was obtained. Confidentiality was maintained by not disclosing the personal information of the participants and by only using the responses found from the interview for the research. Each and every participant was given the right to not participate or to discontinue from the interview whenever they felt uncomfortable. During data collection, the COVID-19 prevention protocol was strictly followed.

## Results

### Sociodemographic characteristics of the study participants

Of the total 408 samples, 396 participants were included in the study with a response rate of 97.06%, and from these total 396 study participants, 194 were cases and 202 controls. Based on the descriptive analysis carried out, 277 (69.9%) of the participants were men, of which 137 were cases and 140 were controls. The age range of the participants was 18 ± 6 years, with a mean and standard deviation (±SD) of 36.06 ± 9.842 years. Only 86 (21.7%) participants had a higher educational level, from which 58.1% were cases and 41.9% were controls, whereas 279 participants were living in the urban areas (70.5%), out of whom 141 (50.5%) were controls and 138 (49.5%) were cases. Nearly 90.7% of the participants are currently living with their family, of whom 178 (49.6%) were cases and 181 (50.4%) were controls. Around one-third, 115 (29%) of the study participants, were married, of whom 54 were cases and 61 were controls. Out of 396 participants, only 141 (35.6%) were found to be currently employed, with only 67 (47.5%) being cases. Around 50% of the total participants started facing financial problems, and from those participants, half of them were cases. The median average monthly income of participants was 5,197.50 ETB with an interquartile range of 3,500–8,350 ETB, as shown in Table 1.

**Table 1:** Sociodemographic characteristics of patients with schizophrenia at the Amanuel Mental Specialized Hospital, Addis Ababa, Ethiopia (*n* = 396).

**Variables**	**Categories**	**Case**		**Control**	
		**Frequency**	**Percentage%**	**Frequency**	**Percentage %**
Sex	Men	137	49.45	140	50.54
	Women	57	47.89	62	52.10
Age category in year	18–24	13	36.11	23	63.88
	25–31	55	55.0	45	45.0
	32–38	61	52.1	56	47.9
	39–45	34	42.5	46	57.5
	46–52	18	47.4	20	52.6
	53–59	6	46.2	7	53.8
	≥60	7	58.3	5	41.7
Educational status	No formal education	27	41.5	38	58.5
	Primary	66	51.6	62	48.4
	Secondary	51	43.6	66	56.4
	Diploma and above	50	58.1	36	41.9
Marital status	Married	54	47.0	61	53.0
	Single	114	50.7	111	49.3
	Divorced	20	52.6	18	47.4
	Widowed	6	33.3	12	66.7
Religion	Orthodox	69	48.6	73	51.4
	Muslim	82	55.4	66	44.6
	Protestant	38	39.6	58	60.4
	Others	5	50.0	5	50.0
Residency	Urban	138	49.5	141	50.5
	Rural	56	47.9	61	52.1
Living arrangement	With family	178	49.6	181	50.4
	Alone	16	43.2	21	56.8
Occupational status	Unemployed	127	49.8	128	50.2
	Employed	67	47.5	74	52.5
Average monthly income	1000–3499	31	32.0	66	68.0
	3,500–5,999	67	48.9	70	51.1
	6,000–8,499	34	51.5	32	48.5
	8,500–10,999	42	70.0	18	30.0
	11,000–13,499	9	56.3	7	43.8
	13,500–15,999	4	44.4	5	55.6
	≥16,000	7	63.6	4	36.4
Facing financial problem	No	95	48.0	103	52.0
	Yes	99	50.0	99	50.0

^*^Other; catholic, Adventist and wakefata.

### Clinical and behavioral characteristics of study participants

The finding showed that 188 (47.5%) participants had a history of substance use in the past 12 months and 105 (55.9%) were cases. The mean ± SD age for first-time substance use was 24.1 ± 6.647 years, with the youngest and oldest ages starting to use substance being 12 and 45 years, respectively. Commonly used substances were Khat (26.3%), cigarette (14.3%), and alcohol (13.0%), in addition to those having a history of substance use for the past 1 year, and 117 (62.2%) of them have a history of using more than one substance.

Regarding medication adherence, only 50 (12.6%) of the individuals, who were found to have good adherence according to the score of MARS-10 and 22 (44%), were cases. The data also showed 21 (5.3%) individuals who were reported of having a known comorbid chronic or neurological illness, as shown in Table 2.

**Table 2:** Clinical and behavioral characteristics of patients with schizophrenia at the Amanuel Mental Specialized Hospital, Addis Ababa, Ethiopia (*n* = 396).

**Variables**	**Categories**	**Case**		**Control**	
		**Frequency**	**Percentage%**	**Frequency**	**Percentage %**
Co–morbid other illnesses	No	186	49.6	189	50.4
	Yes	8	38.1	13	61.9
Current substance use	No	89	42.8	119	57.2
	Yes	105	55.9	83	44.1
Age of first substance use	12–15 years	5	45.5	6	54.5
	16–19years	24	53.3	21	46.7
	20–23 years	17	48.6	18	51.4
	24–27 years	25	58.1	18	41.9
	28–31 years	19	57.6	14	42.4
	≥32 years	15	71.4	6	28.6
Medication adherence	Good	22	44.0	28	56.0
	Poor	172	49.7	174	50.3

### Psychosocial characteristics of the study participants

From the total number of participants, only 7 (28.6% cases and 71.4% controls) and 13 (38.5% cases and 61.5% controls) patients were found to have perceived criticism and emotional involvement by their family members, respectively. A total of 170 (42.9%) participants had at least one stressful experience in the past 12 months, of which 90 (52.9%) were cases and 4.5% were controls, as shown in Table 3.

**Table 3:** Psychosocial characteristics of patients with schizophrenia at the Amanuel Mental Specialized Hospital, Addis Ababa, Ethiopia (*n* = 396).

**Variables**	**Categories**	**Case**		**Control**	
		**Frequency**	**Percentage%**	**Frequency**	**Percentage %**
Perceived criticism	High Perceived criticism	2	28.6	5	71.4
	Low Perceived criticism	192	49.4	197	50.6
Emotional involvement	High involvement	5	38.5	8	61.5
	Low involvement	189	49.3	194	50.7
Life threatening in last 1 year	No experienced	104	46.0	122	54.0
	Had an experienced	90	52.9	80	47.1
Social support	Poor social support	110	61.1	70	38.9
	Strong social support	84	38.9	132	61.1

### Social support and relapse

From the participants, 61.1% of the cases were assessed to have poor social support, whereas by analyzing the data by the chi-square goodness-of-fit test, 110 of the cases and 70 of the controls showed a poor social support system.

In the binary logistic regression analysis, educational status, average monthly income, substance use, Khat users, age of first substance use, life-threatening experience, and social support showed a statistical significance (*p* < 0.05). However, both in the backward likelihood analysis and in binary logistic regression, only social support was found to be statistically significant (*p* < 0.001, AOR = 3.102, CI (95%) = (1.985–4.848). This means that the odds of having symptom relapse were 3.10 times higher for those patients with poor social support when compared to those with good social support, as shown in Table 4.

**Table 4:** Factors associated with relapse in of patients with schizophrenia at the AMSH, Addis Ababa, Ethiopia (*n* = 396).

**Variables**		**Relapse**			
		**Case**	**Control**	**COR (CI 95%)**	**AOR (CI 95%)**
Educational status	No formal education	27	38	0.51 (0.27–0.98)^**^	0.96(0.47–1.98)
	Primary	66	62	0.77 (0.44–1.33)	0.89 (0.47–1.64)
	Secondary	51	66	0.56 (0.32–0.98)^**^	0.57 (0.31–1.05)
	Diploma and above	50	36	1	
Average monthly income	1000–3499	31	66	0.27 (0.07–0.99)^**^	0.35 (0.09–1.34)
	3,500–5,999	67	70	0.57 (0.15–1.95)	0.81 (0.22–3.03)
	6,000–8,499	34	32	0.61 (0.16–2.27)	1.08 (0.27–4.29)
	8,500–10,999	42	18	1.33 (0.35–5.13)	2.37(0.58–9.65)
	11,000–13,499	9	7	0.74 (0.15–3.55)	1.43 (0.28–7.41)
	13,500–15,999	4	5	0.46 (0.08–2.76)	0.64(0.10–4.14)
	>16,000	7	4	1	
Current substance use	No	89	119	1	
	Yes	105	83	1.69^**^ (1.14–2.52)	1.49 (0.97–2.29)
Age of first time substance was used	12–15 years	5	6	1	
	16–19 years	24	21	1.53 (0.80–2.92)	0.44 (0.08–2.29)
	20–23 years	17	18	1.26 (0.62–2.59)	0.47(0.14–1.63)
	24–27 years	25	18	1.86 (0.96–3.61)^*^	0.44 (0.12–1.61)
	28–31 years	19	14	1.82 (0.86–3.82)^*^	0.47 (0.14–1.66)
	>32 years	15	6	3.34 (1.25–8.96)^*^	0.50 (0.14–1.84)
Social support	Poor	110	70	2.47 (1.65–3.71)^**^	3.10 (1.99–4.85)^**^
	Strong	84	132	1	
Stressful life experience in 1 year	None	104	122	1	
	Had stressful event	90	80	1.32^*^(0.89–1.97)	1.07 (0.68–1.67)

1 = A constant or reference (

* , P < 0.05, and

** , P < 0.01); COR, Crude odds ratio; AOR, Adjusted odds ratio.

### Correlation of social support and other relapse determinants

To determine the indirect relation of other determinants with relapse, a bivariate analysis was also conducted to identify the correlation between social supports and relapse determinants like current substance use, medication adherence, perceived criticism, emotional involvement, financial problems, and stressful life events. Variables, like emotional involvement, stressful experience, and facing financial problems, had a correlation with social support, as shown in Table 5.

**Table 5:** A correlation of other relapse determinants with social support in patients with schizophrenia at the AMSH, Addis Ababa, Ethiopia (*n* = 396).

**Variables**		**Social support**
Current substance use	Correlation	−0.09
	*p*–value	0.05
Perceived criticism	Correlation	0.07
	*p*–value	0.17
Emotional involvement	Correlation	−0.11^*^
	*p*–value	0.03
Stressful life experience	Correlation	−0.18^**^
	*p*–value	0.00
Medication adherence	Correlation	0.04
	*p*–value	0.49
Facing financial problem	Correlation	−0.25^**^
	*p*–value	0.00

* Correlation is significant at the 0.05 level (2–tailed).

** Correlation is significant at the 0.01 level (2–tailed).

## Discussion

This study assesses the relationship between social support and schizophrenia relapse and revealed that the majority of participants among cases (61.1%) of those poor social supports had schizophrenia relapse. Participants scoring themselves with the social support scale for the assessment of poor social support in cases were higher as compared to those in the control group, which implies that those case participants who had poor social support were at higher risk for relapse.

Despite this finding, still, a significant number of cases, nearly two-thirds of patients with schizophrenia, who had poor social support, had schizophrenia relapse, which might be attributed to poor social support, schizophrenic patients with emotional expression problems, lack of support to mobilize and bring the patient up to health service, lack of family follow-up of their medication adherence, financial problems, and facing stressful events.

The study reported 61.1% of the cases with poor social support, which was higher as compared to the study done in Jimma, Ethiopia, 26.8% (12). This might be because of the difference in study design since this study used the case-control study design, whereas a cross-sectional study was used in the study done in Jimma (12). The other possible difference might be the geographical and cultural differences in the study area or tools used to assess social support.

Poor social support might increase the risk of relapse by increasing social withdrawal, slowness, underactivity, and apparent lack of motivation, which can highly affect the functionality both socially and occupationally.

Although medication adherence is viewed as the most important determinant of relapse and poor social support can affect medication adherence, this study did not show any significant association between social support and medication adherence, which was contrary to the study done in Tanzania (13). This difference might have arisen because of differences in the study design where the later study should have used a qualitative study design for the exploration of ideas and forming a hypothesis. Therefore, the study cannot test the hypothesis but merely conclude that there was a relationship. The other possible reason for the difference between the present study and that of the study conducted in Tanzania might pertain to the sample size, which can affect the result inference and randomization. In addition, the methods of data collection used by the Tanzanian (13) study were in-depth interviews, which are a way of exploration to show the association of the variables.

The assumption behind good social support decreasing relapses by increasing medication adherence might be due to social support. Steps taken by the social support group can increase the financial stability of the patients, which can help in affording medication and other treatment modalities, in motivating the patients to stick to the treatment plan and take their medication as prescribed, and in increasing close follow-up at home if the patient is taking the medication prescribed or in remembering the patient when he/she forgets to take his/her medication, because that is the main reason for the poor adherence (13).

The present study also showed that there was a negative relation between financial problems, emotional involvement, and stressful life experiences. This negative relation can be interpreted as follows: the increase in social support can decrease issues in financial problems, the emotional involvement of the family, and stressful life experiences and vice versa, which are all viewed as important determinants of relapse in patients with schizophrenia.

Hence, the majority of the participants are unemployed, so having a good social support system can help them bringing about financial stability. In other words, it is believed that the source of finance for the patients are caregivers or families of the patients; therefore, if the social support is poor, the patients might face financial inability or financial instability, which can trigger high financial stress in them. This situation in turn provokes a high rate of relapse in patients with schizophrenia. Furthermore, patients with schizophrenic who have good family support have a lower chance of having a relapse, solely because family support is offered in different ways: affording medication, psychological support, and limiting all the factors, as well as the family bringing the patients to the health facility, even if there is limited health facility in Ethiopia (25).

The other dimension in which social support might be beneficial for patients with schizophrenia in reducing the risk of relapse pertains to the decrease in family emotional expressions, which can be highly stressful for patients. Strong social support might help in decreasing the blame, shouting and overall hostility of the family toward the patient, and having a good family attitude (emotional expression) to reduce the stigma, and the patients should develop insights into what is important for reducing the relapse in patients with schizophrenia (26).

However, the benefit of social support in decreasing relapse is thought to be related to the occurrence and the coping mechanism of stressful life events of patients. Social support is known to decrease the sense of loneliness and isolation, whereas it increases the ability of individuals to better cope with and manage stressful situations. Therefore, it can help patients to have a support system on which they can rely while they are in a stressful situation and develop a better understanding to cope with and manage the stressful situation. It also helps them to have a sense of acceptance by others and of not being left alone to face stressful situations and even their disorder (27, 28).

## Strengths and limitations of the study

The strengths of this study are that it clearly shows the association of social support with relapse with other controlling factors. The use of a case-control study design and standardized tools for data collection is a major strength. However, the cause of recall bias is a limitation.

## Conclusion

This study showed that there was a high relationship between poor social support and the occurrence of relapse in patients with schizophrenia. Poor social support was also found to be high in cases when compared to controls. Social support also showed a negative association with other variables, which are believed to be predictors of relapse in patients with schizophrenia. Better support groups need to be formed for individuals suffering from severe mental disorders, particularly for patients with schizophrenia and patients recovering from schizophrenia. It is necessary to thoroughly include psychosocial interventions in the maintenance phase of the management plan to treat patients with schizophrenia. The social support system should be strictly followed to decrease the patients' relapse rate since it is likely to be neglected.

## Data availability statement

The original contributions presented in the study are included in the article/supplementary material, further inquiries can be directed to the corresponding author.

## Ethics statement

The studies that included human participants were reviewed and approved by, Institutional Health, Research Ethics Review Committee of the College of Medical and Health Sciences, University of Gondar. The patients/participants provided their oral and written informed consent to participate in this study.

## Author contributions

TS was involved from inception to the design acquisition, analysis, interpretation of data, and drafting and editing of the manuscript. YM, KN, and TA were the co-authors who participated in the review of the article, tool evaluation, interpretation, and critical review of the draft manuscript. All authors read and approved the final manuscript.

## Funding

The University of Gondar has funded the data collection of this study. The funders had no role in the design of the study, analysis, interpretation, and publishing of the manuscript.

## Conflict of interest

The authors declare that the research was conducted in the absence of any commercial or financial relationships that could be construed as a potential conflict of interest.

## Publisher's note

All claims expressed in this article are solely those of the authors and do not necessarily represent those of their affiliated organizations, or those of the publisher, the editors and the reviewers. Any product that may be evaluated in this article, or claim that may be made by its manufacturer, is not guaranteed or endorsed by the publisher.

## Abbreviations

DSM 5: Diagnostic and Statistical Manual of Mental Disorders Version 5
FEICS: Family Emotional Involvement and Criticism Scale
LTE: List of Life Threatening Experiences
MARS-10: Medication Adherence Rating Scale-10
WHO: World Health Organization.
